# Experimental Study on the Effect of Polycarboxylate Superplasticizer on the Performance of Cement-Based Grouting Materials

**DOI:** 10.3390/ma17143620

**Published:** 2024-07-22

**Authors:** Zhijie Yu, Shujie Liu, Jiwei Zhang, Wen He, Qinghao Tian, Le Tian, Jinze Sun

**Affiliations:** 1 China Coal Research Institute, Beijing 100013, China; yuzj2020@163.com (Z.Y.); 13681342073@163.com (S.L.); 15618536658@163.com (J.S.); 2 National Engineering Research Center of Deep Shaft Construction, Beijing 100013, China; 3 Beijing China Coal Mine Engineering Company Ltd., Beijing 100013, China; wenhe1006@126.com (W.H.); 13811889035@163.com (L.T.); 4 School of Civil and Resource Engineering, University of Science and Technology Beijing, Beijing 100085, China; 5 China Coal Technology Engineering Group, Beijing 100013, China; tianqinghao1997@163.com

**Keywords:** cement-based grouting materials, polycarboxylate superplasticizer, mechanical properties, microstructure

## Abstract

Polycarboxylate superplasticizers BMC-L and BMC-S were utilized as modifiers in the formulation of novel cement-based grouting materials. Indoor tests were conducted on 32 groups of cement slurries, varying by water–cement ratio (0.5:1 and 0.6:1) and modifier content (0, 2‰, 4‰, 6‰, 8‰, 10‰, 12‰, and 14‰), to test their density, funnel viscosity, water separation rate, and stone rate. Four groups of slurry modified with BMC-L were selected as the preferred slurry, and the apparent viscosity test, uniaxial, and triaxial compression test of the slurry stone body were conducted. The study investigated the influence of BMC-L on various properties of the slurry, including its apparent viscosity, uniaxial compressive strength, stress–strain relationships, shear strength parameters, and elastic modulus. Ultimately, the pore structure and phase composition of the slurry stone body were detected by Nuclear Magnetic Resonance (NMR) and X-ray Diffraction (XRD), and the impact of BMC-L on slurry performance was examined from a microstructural perspective. Results indicate that the two polycarboxylate superplasticizers exert minimal influence on the density and water separation rate of the slurry. Within the effective incorporation range of the polycarboxylate superplasticizer, increasing the dosage correlates with a decrease in both the stone rate and viscosity of the slurry. BMC-L significantly enhances the mechanical properties of the slurry stone body by promoting more complete cement hydration and reducing porosity. The uniaxial compressive strength of slurry stone body with a 6 ‰ BMC-L dosage reached 29.7 MPa after 7 days and 38.5 MPa after 28 days of curing, representing increases of 118.4% and 64%, respectively, compared to masonry with 0 BMC-L dosage. The shear strength parameters and elastic modulus of the slurry stone body also showed corresponding increases.

## 1. Introduction

Grouting technology refers to formulating suitable cementitious materials into a targeted slurry, which is then injected, either directly or indirectly, into the geotechnical body using pressurized equipment. This process aims to alter the mechanical distribution and structural composition of the geotechnical body. Subsequent solidification of the slurry with the existing formation creates a composite body, enhancing the physical and mechanical properties to achieve waterproofing and reinforcement objectives [1]. Currently, grouting technology is extensively employed in managing water damage and reinforcing surrounding rock in mines, along with applications in tunnel and foundation engineering. This method plays a crucial and irreplaceable role in resource safety development, geological disaster management, and the reinforcement of underground surrounding rock [2,3,4,5,6]. Current research on grouting technology primarily concentrates on grouting theory, materials, and techniques. These studies are particularly significant for advancing green, efficient, and safe practices, with a special emphasis on the development and improvement of grouting materials.

Grouting material constitutes a critical component of grouting technology and is a pivotal factor in determining the effectiveness of grouting [7,8]. Since the advent of grouting technology, a diverse range of grouting materials has emerged, primarily categorized into inorganic and organic types. Cement slurry, comprising primarily cement mixed with minor additives and water, represents the earliest and most prevalent inorganic grouting material. This mixture is prepared and injected in a single-fluid manner. The advantages of cement slurry include its abundant availability, low cost, high compressive strength, effective seepage resistance, non-toxicity, simplicity of the process and equipment, and ease of operation [9,10]. However, the disadvantages of cement slurry include a prolonged setting time, low initial strength, slow strength development, susceptibility to water separation, poor injectability, and vulnerability to being washed away by groundwater [11]. Consequently, to enhance the performance of cement slurry in practical engineering applications, various modifiers—including quick-setting agents, early strength agents, suspending agents, lime, graphene oxide, and nano-additives—are commonly incorporated.

In recent years, extensive research has been conducted by scholars on the influence of various modifiers on the performance of cement-based grouting materials. Jia et al. [12] incorporated polypropylene short fibers of 3 mm and 6 mm lengths into cement grouting materials and assessed their impact on fluidity, mechanical properties, drying shrinkage rate, and pore structure. The research revealed that polypropylene fibers enhance toughness, reduce water loss, and diminish drying shrinkage. Specifically, the use of 0.9 vol% 3 mm polypropylene fibers resulted in a 38% increase in the 28-day flexural strength of the grouting material and a 22% reduction in the drying shrinkage rate. Hu et al. [13] incorporated two modifiers—early-strength and high-strength grouting modifiers—into cement-based grouting materials. They evaluated the impact of these modifiers on setting time, fluidity, plastic viscosity, and yield stress under varying water-to-cement ratios, ultimately focusing on the rheological properties of the materials. Cheng et al. [14] incorporated varying amounts of graphene oxide into cement-based grouting materials and assessed the resultant changes in crystal structure, mechanical properties, and self-shrinkage characteristics of the modified materials. Yuan et al. [15] introduced stabilizers and reinforcing agents into cement slurry, achieving a reduction in water separation rate from 21% to 5% for slurries with a 1:1 water–cement ratio. This modification maintained the compressive strength comparable to that of untreated slurries and significantly enhanced overall performance. Wang et al. [16] developed a fast-hardening cementitious grouting material using sulphur–aluminate cement and investigated the influence of nano-SiO_2_ on the fluidity and mechanical properties of the grouting materials across various water–cement ratios. The study found that adding 1.0% nano-SiO_2_ increased the compressive strength of the grouting material by up to 44% without sacrificing fluidity. Shen et al. [17] explored the impact of mineral admixtures—including fly ash, limestone powder, and silica fume—on the fluidity, rheological properties, and strength of cement-based grouting materials. Compared to pure cement grouting, the initial fluidity and 60 min fluidity of grouting materials with 40 wt% fly ash decreased by 35.5% and 53.8%, respectively.

Polycarboxylate superplasticizers are a type of admixture employed in concrete modification that disperses cement particles, reduces unit water consumption, and enhances the fluidity of the concrete mix. In recent years, advancements in the research and development of polycarboxylate superplasticizers have led scholars to explore their use in modifying grouting materials. Costa A. Anagnostopoulos [18] utilized polycarboxylate superplasticizer (PCE) to modify cement slurry and investigated its effects on the rheological properties, mechanical strength, and water precipitation rate of the slurry through a series of experiments. The study found that the addition of PCE induced shear thickening in the slurry. For slurries with water–cement ratios of 0.4 and 0.5, the incorporation of PCE significantly increased the strength of the slurry stone body. However, in slurries with a water–cement ratio of 0.3, high doses of PCE reduced the early strength development and resulted in limited final strength growth. Puertas [19] examined the impact of polycarboxylate (PC) admixtures on the mechanical, mineralogical, microstructural, and rheological properties of Portland cement slurries. The research demonstrated that PC admixtures delay the initial hydration reaction of cement, reduce the maximum yield stress of the slurry by 70%, and alter the microstructure of the slurry stone body, albeit with minimal effect on the strength of the stone body. Zhang et al. [20] investigated the effect of polycarboxylate superplasticizers on the performance of a fine tailings filler slurry composed of cement and fine tailings. The study found that the addition of polycarboxylate superplasticizers induced shear thinning in the slurry, reducing the early strength of the slurry stone by 10.29% but ultimately increasing the strength by 10.51%. At present, there is still a lack of research and literature on the modification of grouting materials using polycarboxylate superplasticizers, especially in the quantitative analysis of the micropores and phase composition of slurry stone bodies. Further research is needed.

To enhance grouting efficiency, fluidity of cement slurry at low water–cement ratios, and slurry stability, and to offer scientific guidance on material proportioning, this study employs polycarboxylate superplasticizers BMC-L and BMC-S as modifiers in the development of new cement-based grouting materials. Utilizing indoor testing, this study tested the basic performance and mechanical performance of cement slurries across different formulations varying in water–cement ratios and polycarboxylate superplasticizer content, exploring the impact of polycarboxylate superplasticizers on the mechanical properties of cement-based grouting materials. Additionally, the study examines the evolution of the microscopic pore structure and phase composition of the slurry stone body with varying dosages of polycarboxylate superplasticizer. This provides a detailed analysis of the effects of the polycarboxylate superplasticizer on cement-based grouting materials at the microstructural level.

## 2. Materials and Methods

### 2.1. Materials

The cement utilized in this study was ordinary silicate P.O.42.5, with its primary components detailed in Table 1.

Two forms of polycarboxylate superplasticizer were used: BMC-L and BMC-S. The acronym “BMC” represents the author’s institution, while “L” and “S” denote the liquid and solid states of the superplasticizer, respectively. Their properties are presented in Table 2.

### 2.2. Methods

**Figure 1:**
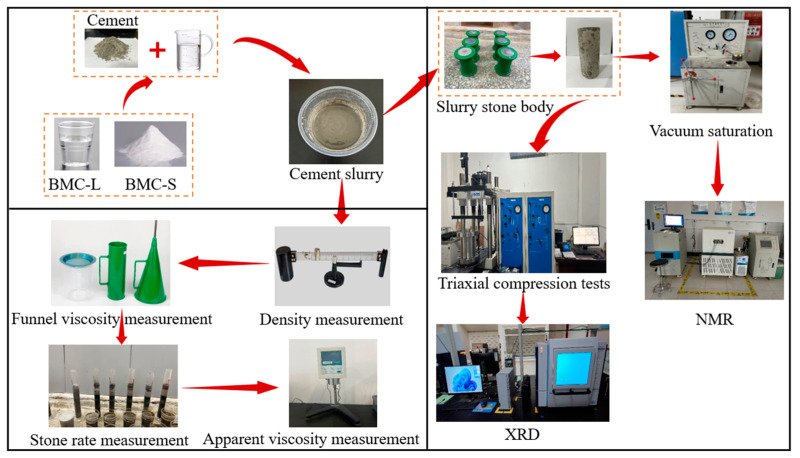
Experimental procedure.

This paper investigates the impact of polycarboxylate superplasticizer on the basic and mechanical performance, microstructure, and phase composition of cement-based grouting materials. The experimental procedure is outlined in Figure 1. The basic performance of the slurry was assessed using a slurry hydrometer, a funnel viscometer, a 100 mL measuring cylinder, and a rotary viscometer. Mechanical performance was evaluated with a low-temperature and high-pressure triaxial test system. The distribution of water within the saturated sample was examined using nuclear magnetic resonance (NMR), enabling the characterization of the micropore distribution within the slurry stone. The phase composition of the slurry stone was quantitatively analyzed through X-ray diffraction (XRD).

#### 2.2.1. Sample Preparation

1. Cement slurry preparation

Two kinds of cement slurry were prepared with water–cement ratios of 0.5:1 and 0.6:1, respectively. All grouts were mixed using a three-blade paddle high-rotating mixer. The required cement and the corresponding amount of mixing water were accurately weighed based on the predefined ratios. The mixing pot and blade were moistened without leaving standing water. The cement was poured into the mixing pot, the mixer was turned on, and the mixing water was added concurrently, ensuring the process was completed within 10 s. The addition of superplasticizers in the cement slurry was performed using the delayed addition method. Particularly, after 5 min of stirring cement and mixing water in the mixer, the predefined dosage of superplasticizers was added to the cement slurry. Then, continuous stirring was performed for a total time of at least 2 min. The selection of this method for adding superplasticizers is because the delayed addition of superplasticizers in cement suspensions significantly enhances the efficacy of its dispersing power in comparison to the direct addition. The dosages of each polycarboxylate superplasticizer should vary at concentrations of 0, 2‰, 4‰, 6‰, 8‰, 10‰, 12‰, and 14‰ relative to the cement mass.

2. Slurry stone body preparation

The prepared cement slurry was poured into molds with a diameter of 50 mm and a height of 100 mm. The molds were then placed on a concrete vibration table to expel air from the slurry using mechanical vibration. After thorough vibration, the molds were transferred to a standard concrete curing room maintained at a temperature of 20 ± 2 °C and a relative humidity of ≥95%. After 24 h, the molds were removed, and the samples were placed in the same environment for further curing periods of 3 days, 7 days, and 28 days, respectively. Post-curing, the longitudinal wave velocity of the specimens was measured using an KON-NM-4A non-metallic ultrasonic detection analyzer produced by Wuhan Corey Instrument Equipment Co., Ltd. in Wuhan, China. This velocity served as the criterion for screening, with specimens exhibiting similar wave velocities selected to minimize the test dispersion.

#### 2.2.2. Testing Procedures

1. Basic slurry performance testing and formulation screening.

Density measurements of the slurry were conducted using ANY-1 Mud Hydrometer, produced by Suzhou Zhongtong Testing Instrument Co., Ltd. in Suzhou, China with a measurement range of 0.96–3.0 g/cm^3^. The sample of cement slurry was filled into the cup of the Mud Hydrometer, then covered, and any overflow was removed. The instrument was then placed on its support and adjusted using the vernier until horizontal. The scale reading on the left side of the vernier provided the relative density of the slurry.

Viscosity was initially assessed using ANY-1 Funnel Viscometer produced by Suzhou Zhongtong Testing Instrument Co., Ltd. in Suzhou, China. A screen was placed over the funnel, which was then covered at the lower end with fingers. A 700 mL sample of the cement slurry was poured through the screen into the funnel. Below this, a 500 mL measuring cylinder was positioned. Timing commenced upon removing the fingers and stopped when the slurry reached the top of the cylinder. The stopwatch reading indicated the funnel viscosity of the slurry, expressed in seconds.

Water separation and stone rates were determined using a 100 mL measuring cylinder. The cylinder was filled with 100 mL of cement slurry, sealed with plastic wrap, and allowed to stand for 2 h. The proportion of water separated from the slurry was then calculated to ascertain the water separation rate. Following solidification into a stone body, the stone-to-slurry volume ratio was computed to determine the stone rate.

Through analyses of slurry density, funnel viscosity, 2 h water separation rate, and stone rate, the impact of two polycarboxylate superplasticizers on slurry fluidity and stability was assessed. This facilitated the identification of the optimal superplasticizer and the formulation of the slurry exhibiting superior performance.

2. Slurry apparent viscosity and mechanical performance test of slurry stone body

Following the selected slurry formula, the cement slurry was prepared anew, and its apparent viscosity was measured using a DHJ-5S/8S rotary viscometer produced by Ningbo Lawson Smarttech Co., Ltd. in Ningbo, China.

In accordance with the “Technical code for application of cementitious grout” (GB/T 50448-2015) [21], uniaxial compression tests were conducted using the FRTX-1000 low-temperature and high-pressure servo-controlled rock triaxial test system produced by GCTS in San Jose, CA, USA on slurry stone body cured for 3, 7, and 28 days. Additionally, triaxial compression tests on the 28-day cured samples were performed with confining pressures set at 100 KPa, 200 KPa, and 300 KPa.

3. Analysis of microscopic pore characteristics and phase composition of the slurry stone body.

The microscopic pore characteristics of the slurry stone body were analyzed using the NIUMAG MesoMR12-060H-1 CORE MRI system produced by Suzhou Niumag Analytical Instrument Corporation in Suzhou, China. Prior to the NMR analysis, the slurry stone specimens required saturation with water. This study utilized the vacuum forced saturation method, recognized for its superior saturation effects, employing the NM-V vacuum pressurized saturation device for this purpose. Following 48 h of forced saturation, the specimens were removed and encased in plastic wrap in preparation for the NMR test.

Phase composition of the slurry stone body was determined using Bruker D2 Phase X-ray Diffractometer produced in Bruker, Berlin, Germany. Samples were first crushed with select debris ground using a mortar. After grinding, the samples were prepared using the positive pressure method and put into the chamber for XRD.

## 3. Results and Discussion

### 3.1. Slurry Density

**Figure 2:**
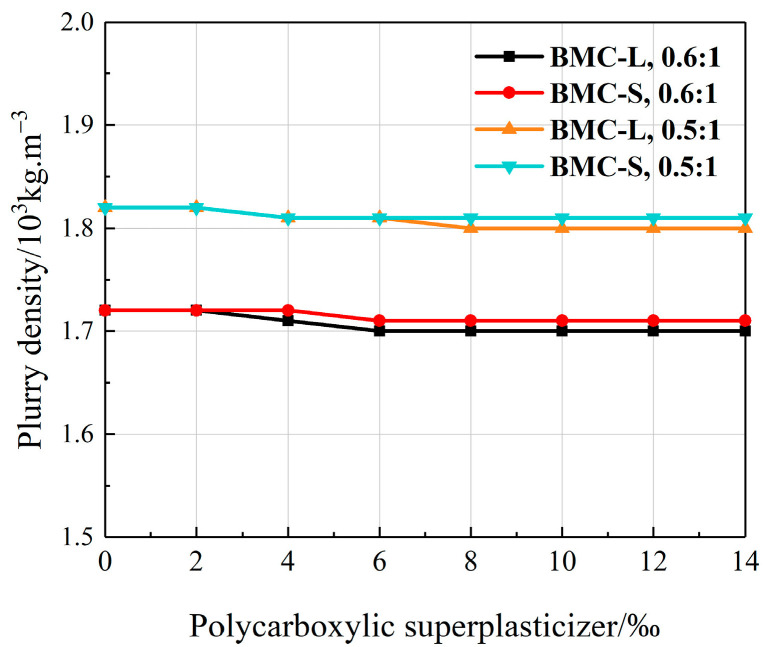
Effect of polycarboxylate superplasticizer dosage on slurry density.

Slurry density significantly influences the pumpability of cement slurry during the grouting process. Excessively high slurry density diminishes fluidity and pumpability, whereas excessively low density increases fluidity, potentially resulting in cement slurry loss and uneven deposition. A relative densitometer measured the density of each slurry test group, with results presented in Figure 2. As illustrated in Figure 2, slurry density exhibits minimal variation with increasing dosages of the two polycarboxylate superplasticizers. Notably, densities fluctuate slightly when the dosage is below 6‰; however, densities stabilize when the dosage exceeds 6‰. This phenomenon may be attributed to the full reaction between polycarboxylate superplasticizer and cement, reaching a saturation point at a dosage of 6‰. Additionally, the figure indicates that the water–cement ratio critically affects slurry density; a higher water–cement ratio correlates with lower density and better pumpability.

### 3.2. Slurry Funnel Viscosity

Viscosity is defined as the measure of internal friction experienced by molecules during liquid flow. In slurries, the magnitude of viscosity directly influences the diffusion radius and critically determines parameters such as grouting pressure and flow rate. As slurry viscosity increases, both its fluidity and diffusion radius decrease correspondingly.

**Figure 3:**
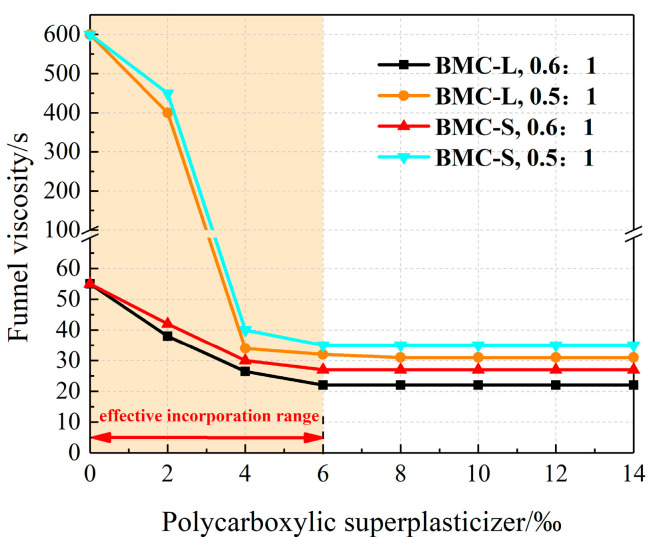
Effect of polycarboxylate superplasticizer dosage on slurry funnel viscosity.

Funnel viscosity for each test slurry was measured using a funnel viscometer, with results detailed in Figure 3. As illustrated in Figure 3, within the 0~6‰ dosage range of two types of polycarboxylate superplasticizers, an increase in dosage significantly reduces the slurry funnel viscosity, which does not follow a linear relationship. Notably, for a water–cement ratio of 0.5:1, the addition of the polycarboxylate superplasticizers resulted in a sudden change in funnel viscosity. When the dosage of polycarboxylate superplasticizer was increased from 6‰ to 14‰, there was negligible change in slurry funnel viscosity, indicating an effective incorporation range of 0~6‰ for the polycarboxylate superplasticizers used in this study. In this range, slurry funnel viscosity decreases with the increase of polycarboxylate superplasticizer content. Within this incorporation range, an increase in polycarboxylate superplasticizer content results in decreased slurry funnel viscosity. In addition, under identical conditions of water–cement ratio and polycarboxylate superplasticizer dosage, BMC-L exhibited lower slurry funnel viscosity compared to BMC-S, highlighting its superior efficacy in reducing viscosity. After adding polycarboxylate superplasticizer, the viscosity of the slurry significantly decreases. This may be because the adsorption layer formed by superplasticizer molecules on the surface of cement particles reduces direct contact between particles, thereby reducing the internal friction and cohesion of the mixture. This makes the cement slurry have lower viscosity and better fluidity at the same water content, which is helpful for pumping and construction of the slurry.

### 3.3. Slurry 2 h Water Separation Rate and Stone Rate

The water separation rate is a critical metric for assessing the stability and pumpability of slurry, significantly influencing the structure of the stone body and the efficacy of grouting reinforcement. A lower water separation rate reduces the tendency for segregation and delamination, enhancing the suitability of the slurry for pipeline transportation and its ability to fill fissures and pores effectively. The water separation rate over a specific duration indicates the slurry’s stability. A slurry that maintains a water separation rate of no more than 5% after standing for 2 h is considered stable.

**Figure 4:**
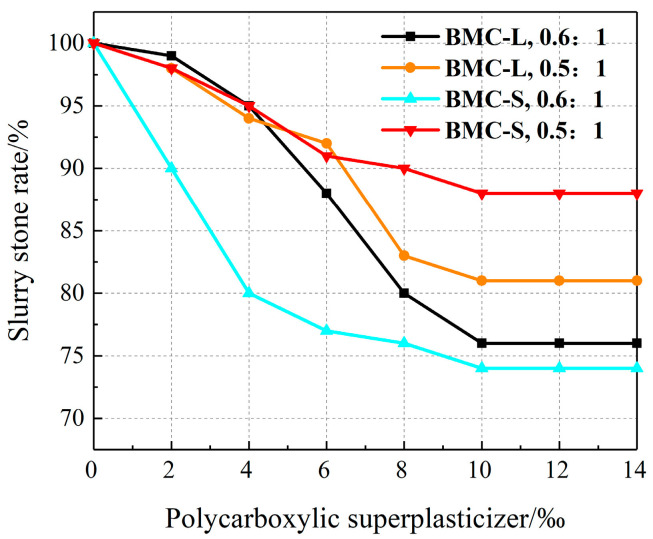
Effect of polycarboxylate superplasticizer dosage on slurry stone rate.

The 2 h water separation rate for each test slurry was determined using a 100 mL measuring cylinder. After the test, the 2 h water separation rate of each group of slurry is 0, which indicates that the two polycarboxylate superplasticizers have almost no effect on the 2 h water separation rate of the slurry, and the slurry is a stabilized slurry after mixing polycarboxylate superplasticizer. Following complete solidification, the stone rate was measured. Figure 4 illustrates the impact of varying dosages of polycarboxylate superplasticizers on the stone rate, showing an initial decrease followed by stabilization as the dosage increases. For a 0.6:1 water–cement ratio slurry, BMC-S more significantly reduces the stone rate than BMC-L. At a 0.5:1 water–cement ratio, the impact of polycarboxylate superplasticizers on the stone rate is comparable within the 0~6‰ range. Beyond this dosage, BMC-L is greater than BMC-S in reducing the stone rate. The reduction in slurry stone rate after the addition of polycarboxylate superplasticizer can be attributed to its effective dispersion effect, which diminishes aggregation among cement particles. This dispersion results from the unique molecular structure of the superplasticizers, particularly the side chains that form an adsorption layer on the cement particle surfaces, enhancing the repulsive forces between them.

### 3.4. Slurry Formulation Selection

To advance the study of how polycarboxylate superplasticizer affects the apparent viscosity of the slurry and the mechanical properties of the slurry stone body, a comprehensive analysis of density, funnel viscosity, 2 h water separation rate, and stone rate for each test slurry was conducted. The relatively optimal slurry formulations were selected for the subsequent slurry apparent viscosity test, uniaxial compression test, triaxial compression experiments, nuclear magnetic resonance test, and X-ray diffraction test.

The density of the tested slurries was more significantly influenced by the water–cement ratio compared to the dosage of the water-reducing agent. Comparative analysis was performed on slurry funnel viscosity across two water–cement ratios, two types of polycarboxylate superplasticizers, and various dosages. The effective dosage range for polycarboxylate superplasticizers was identified as 0~6‰. Notably, slurries with a 0.6:1 water–cement ratio and BMC-L superplasticizer exhibited lower funnel viscosities compared to others. A comparative analysis of the slurry stone rates revealed that mixtures with a 0.5:1 water–cement ratio and BMC-L superplasticizer generally exhibited higher stone rates than others. Within the dosage range of 0 to 6‰ for polycarboxylate superplasticizers, comparing slurry with water–cement ratios of 0.6:1 using BMC-L and 0.5:1 using both BMC-L and BMC-S, the stone rates are relatively similar.

Following a comparison of performance indices among the various slurry groups—distinguished by two water–cement ratios, two types of polycarboxylate superplasticizers, and varying dosages—the slurry formulation with a 0.6:1 water–cement ratio, BMC-L type superplasticizer, and water-reducing agent dosages of 0, 2‰, 4‰, and 6‰ was chosen for subsequent experimental investigations.

### 3.5. Apparent Viscosity of Slurry

**Figure 5:**
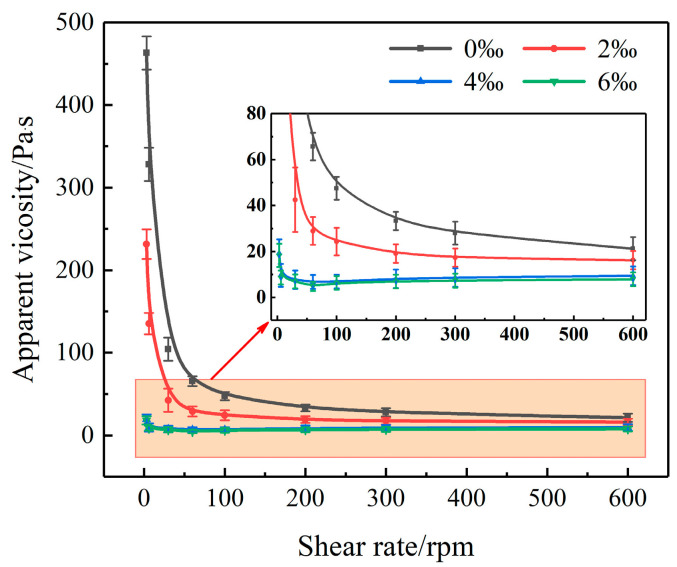
Relationship between apparent viscosity and shear rate with different BMC-L dosages.

The slurry outlined in Section 3.4 is prepared, and its apparent viscosity is measured using a DHJ-5S/8S rotary viscometer produced by Ningbo Lawson Smarttech Co., Ltd. in Ningbo, China. Figure 5 illustrates the relationship between the apparent viscosity of the slurry and the shear rate at various BMC-L dosages. Figure 5 demonstrates that as the shear rate increases, the apparent viscosity of the slurry decreases, exhibiting “shear thinning” behavior where viscosity diminishes with higher shear rates. Compared to formulations without BMC-L, the addition of BMC-L significantly lowers the apparent viscosity of the cement slurry, with greater reductions observed at increasing dosages of BMC-L across various shear rates. At BMC-L dosages of 4‰ and 6‰, the shear rate–apparent viscosity curves nearly coincided, suggesting that at dosages ≥ 6‰, the slurry’s apparent viscosity reaches a minimally low level, beyond which further increases in BMC-L dosage minimally affect the viscosity.

### 3.6. Mechanical Properties of Slurry Stone Body

#### 3.6.1. Uniaxial Compressive Strength

**Figure 6:**
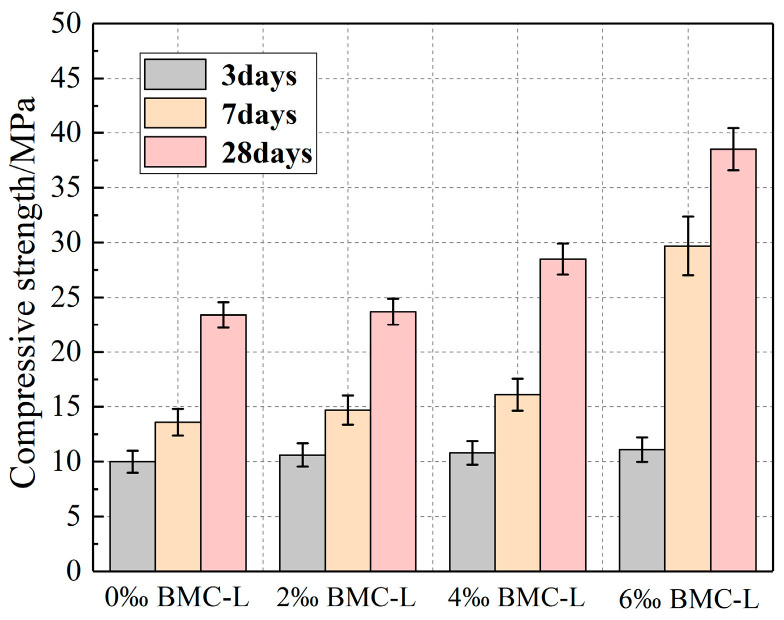
Relationship between BMC-L dosage and compressive strength of the slurry stone body.

The uniaxial compressive strength of the slurry stone body is a crucial mechanical property that significantly influences slurry applications. Figure 6 illustrates the relationship between the uniaxial compressive strength of the slurry stone body and the BMC-L dosage. It is observed that the uniaxial compressive strength of slurry stone bodies increases with the addition of BMC-L, with minimal strength gains at 3 days and significant increases at 7 and 28 days. At a BMC-L content of 6‰, the uniaxial compressive strengths at 7 and 28 days are 118.4% and 64% higher, respectively, than those of slurry without BMC-L. The increase in compressive strength of the slurry stone body is attributed to the use of polycarboxylate superplasticizers, which enhance the uniformity and density of the slurry. The hydration products formed after cement hydration, such as calcium silicate hydrates (C-S-H), are distributed more evenly throughout the slurry and fill its micropores, thus bolstering the mechanical properties of the stone body. Moreover, polycarboxylate superplasticizers delay the initial phase of cement hydration, moderating the dissolution and hydration reactions of cement particles. Consequently, the initial increase in strength of the slurry stone body is relatively modest but significantly greater in the later stages.

#### 3.6.2. Stress–Strain Relationship

**Figure 7:**
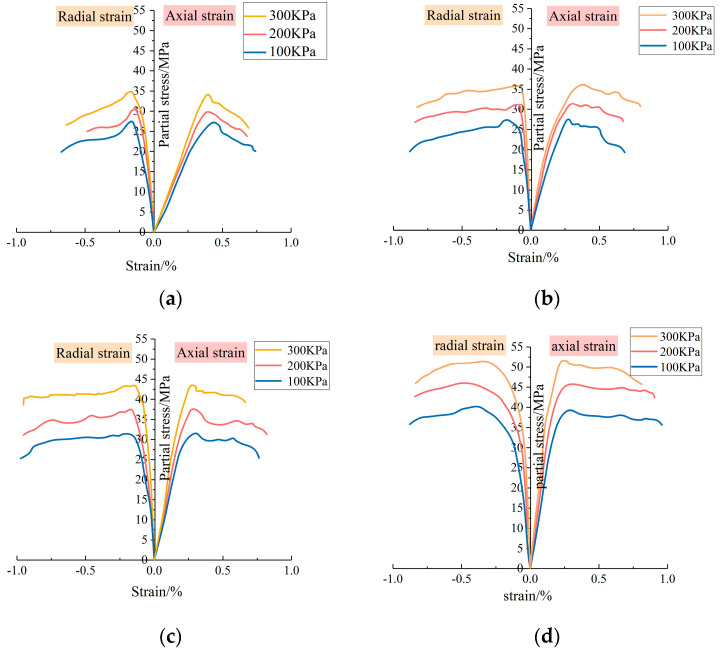
Stress–strain relationship of slurry stone bodies with different BMC-L dosages under different confining pressures: (**a**) BMC-L dosage: 0; (**b**) BMC-L dosage: 2‰; (**c**) BMC-L dosage: 4‰; (**d**) BMC-L dosage: 6‰.

Figure 7 displays partial stress–strain curves for slurry stone bodies with varying dosages of BMC-L, subjected to triaxial compression under different confining pressures. As depicted in Figure 7, the stress–strain curves of slurry stone bodies under various confining pressures follow a similar trend. Initially, stress increases with strain; upon reaching peak stress, it then decreases with further strain increase. Eventually, a sharp decline in stress indicates specimen failure.

The stress–strain relationship in slurry stone bodies with varying BMC-L dosages encompasses three distinct stages: (1) Elastic Deformation Stage: At the beginning of loading, the relationship between partial stress and strain is essentially linear. Axial strains primarily result from the compression of pore spaces between particles. With the axial loading, the particles are gradually compressed, and radial strain at this stage is smaller; (2) Plastic Deformation Stage: As axial and radial strains increase, the slope of the stress–strain curve decreases, indicating a nonlinear relationship and rapidly approaching peak stress. During this phase, internal particle compression and extrusion occur alongside local plastic deformation and cracking within the specimen; (3) Destruction Stage: the partial stress reaches the peak value, and the crack of the slurry stone body continues to expand. Finally, the stone body enters the destruction stage after rupture, and the curve shows a fluctuating downward state. Additionally, the figure illustrates that under a fixed confining pressure, the maximum partial stress in the slurry stone body increases with higher BMC-L dosages. Conversely, at a constant BMC-L dosage, the maximum partial stress escalates with rising confining pressure.

#### 3.6.3. Shear Strength Parameters

To determine the shear strength parameters at various BMC-L dosages, the maximum partial stress served as the criterion for evaluating shear strength. With reference to the relevant triaxial compression test studies, the experimental results were solved by the programming solver tool of Excel 2016 [22] to obtain the cohesion and internal friction angle of each group of specimens, and the collated results are shown in Table 3.

**Figure 8:**
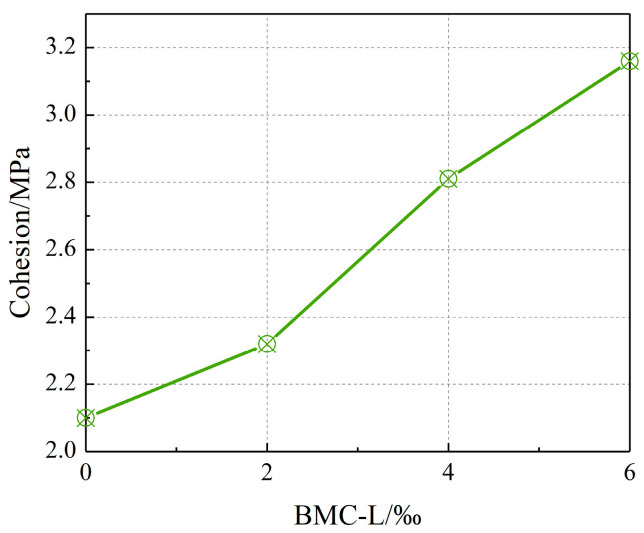
Effect of BMC-L dosage on the cohesion of the slurry stone body.

**Figure 9:**
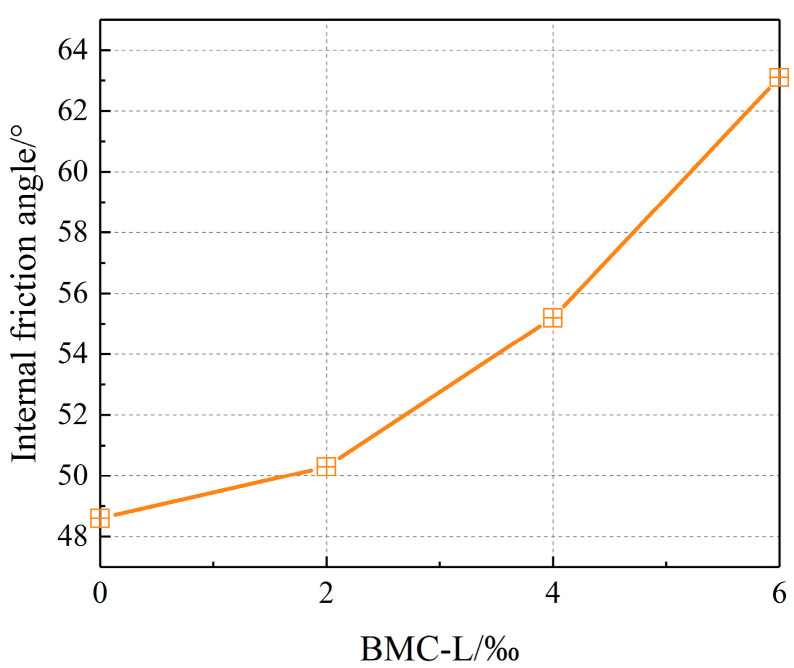
Effect of BMC-L dosage on the internal friction angle of the slurry stone body.

The influence of BMC-L dosage on the cohesion and internal friction angle of the slurry stone body is illustrated in Figure 8 and Figure 9. As depicted in the figures, an increase in BMC-L dosage results in higher cohesion and internal friction angles. Specifically, cohesion escalated from 2.1 MPa to over 3 MPa, marking a maximum increase of 50.4%. Similarly, the internal friction angle rose from approximately 48 degrees to over 60 degrees, achieving a maximum increase of 29.8%. This indicates that BMC-L positively enhances the shear strength of the slurry stone body. This improvement is attributed to BMC-L’s role in dispersing cement particles and expanding their hydration reaction surface area, which facilitates the production of more cement hydration products, leading to a more uniform and denser structure.

#### 3.6.4. Elastic Modulus

The elastic modulus of a slurry stone body characterizes its stiffness, representing the resistance of the stone body to deformation under load. This parameter is crucial for determining the bearing capacity and deformation resistance of the stone body. Referring to the related research, the elastic modulus E is calculated from the slope between the 30% and 70% strength peaks on the stress–strain curve and can be calculated using the following formula:

(1) $$E=\frac{\sigma_{70\%}-\sigma_{30\%}}{\varepsilon_{70\%}-\varepsilon_{30\%}}$$

**Figure 10:**
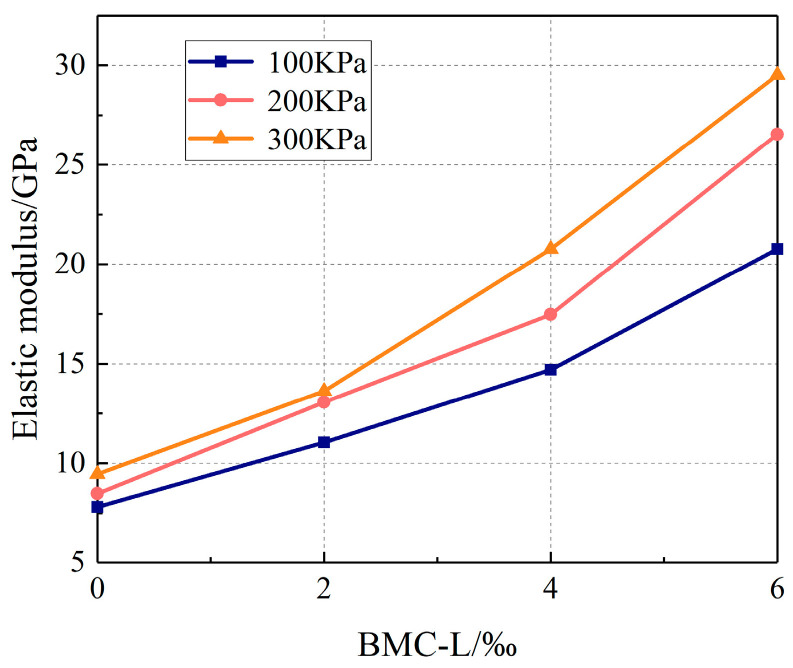
Relationship between the elastic modulus of the slurry stone body and BMC-L dosage.

In the formula, $\sigma_{70\%}$ represents 70% maximum stress, $\sigma_{30\%}$ represents 30% maximum stress, $\varepsilon_{70\%}$ represents strain corresponding to 70% maximum stress, and $\varepsilon_{30\%}$ represents strain corresponding to 30% maximum stress. According to this formula, the elastic modulus of the slurry stone body with different BMC-L dosages under different confining pressures is obtained. Figure 10 illustrates the relationship between the elastic modulus of the slurry stone body and varying dosages of BMC-L. The figure demonstrates that under all tested confining pressures, the elastic modulus increases with higher BMC-L dosages. This suggests that BMC-L improves the microstructure and stiffness of the slurry stone body, thereby increasing its resistance to compressive deformation. At a constant BMC-L dosage, the elastic modulus rises with increasing confining pressure. Notably, at a lower confining pressure (100 KPa), the increase in elastic modulus is relatively slow, whereas, at higher pressures (200 KPa and 300 KPa), it escalates more rapidly with additional BMC-L. This effect is attributed to higher confining pressures compacting the internal pores of the slurry stone body, resulting in a denser structure and, thus, a higher elastic modulus.

### 3.7. Pore Structure and Phase Composition of the Slurry Stone Body

#### 3.7.1. Pore Structure

Recently, low-field nuclear magnetic resonance (NMR) has emerged in the geotechnical field as a rapid, non-destructive technology that provides an intuitive display of detection and can effectively characterize changes in the internal microscopic pore structure of rocks. In the natural state, the spin of the ^1^H nucleus is in a disordered state without magnetization. When the sample is placed in the test chamber of the nuclear magnetic resonance (NMR) instrument, the action of the main magnetic field will make the ^1^H nucleus magnetization phenomenon. The interaction between the spin magnetization of the ^1^H nucleus and the applied magnetic field will produce a signal that can be measured by the instrument, which is the prerequisite for the generation of NMR. The ^1^H nuclear proton leaps from a low-energy state to a high-energy state, a phenomenon known as NMR. It rotates along the direction of the applied magnetic field and releases energy to the outside world. The process of transition from a high-level state to a low-level state is called nuclear magnetic resonance relaxation. The transverse relaxation time decay, or *T*_2_ decay, contains the physical information of most porous samples, so *T*_2_ is the main target of rock NMR detection. According to the mechanism of nuclear magnetic resonance detection, the slurry stone body is subjected to vacuum-forced saturation to ensure that the water is fully filled with the internal pores of the slurry stone body. The *T*_2_ spectra of the slurry stone body are obtained by nuclear magnetic resonance test of the ^1^H nucleus in the water, and the internal pore characteristics and variation rules of the slurry stone body can be obtained by analyzing the *T*_2_ spectra.

The *T*_2_ relaxation time is categorized into surface *T*_2_ relaxation, free *T*_2_ relaxation, and diffusion *T*_2_ relaxation. For porous materials such as the slurry stone body, the relaxation time is mainly affected by the surface *T*_2_ relaxation, and thus, the *T*_2_ can be expressed according to the following formula [23]:

(2) $$\frac{1}{T_{2}}=\rho_{2}\left(\frac{S}{V}\right)$$

In the formula, *T*_2_ represents transverse relaxation time (ms), $\rho_{2}$ represents the surface relaxation strength of *T*_2_ (μm/ms), *S* represents pore surface area (cm^2^), and *V* represents pore volume (cm^3^). It can be seen that the transverse relaxation time *T*_2_ is proportional to the pore-specific surface area (*S*/*V*) and the surface relaxation strength $\rho_{2}$, and Formula (2) can be further simplified to Formula (3):

(3) $$r=\rho_{2}F_{2}T_{2}$$

In the formula, *r* represents the pore radius (μm), *F*_2_ represents the shape aggregation factor, taking value 3 for spherical pores and 2 for columnar pores, and $\rho_{2}$ represents the surface relaxation strength of *T*_2_ (μm/ms); the value can be referred to in Table 4. Li et al. [24] and Zhang et al. [25] suggested a range of values for $\rho_{2}F_{2}$ from 0.01 to 0.15 μm/ms. In this paper, the value of 0.01 μm/ms is taken. This establishes a link between the transverse relaxation time *T*_2_ and the pore radius *r*. It can be seen that the larger the value of *T*_2_, the larger the pore radius, and vice versa.

The pore structure of the slurry stone body includes pore distribution and pore aperture. Pore aperture directly influences the macroscopic strength of slurry stone bodies and is typically classified into micropores, mesopores, and macropores. Currently, there is no standard uniform method for this classification. Table 5 summarizes commonly used methods for classifying pore apertures. It is evident that scholars vary in their definitions of micropores, mesopores, and macropores. In this study, pore apertures are classified according to Yan Jianping’s method: micropores are defined as pores smaller than 0.1 μm, corresponding to *T*_2_ times less than 10 ms; mesopores range from 0.1 μm to 1 μm, with *T*_2_ times between 10 ms and 100 ms; and macropores exceed 1 μm, corresponding to *T*_2_ times greater than 100 ms.

**Figure 11:**
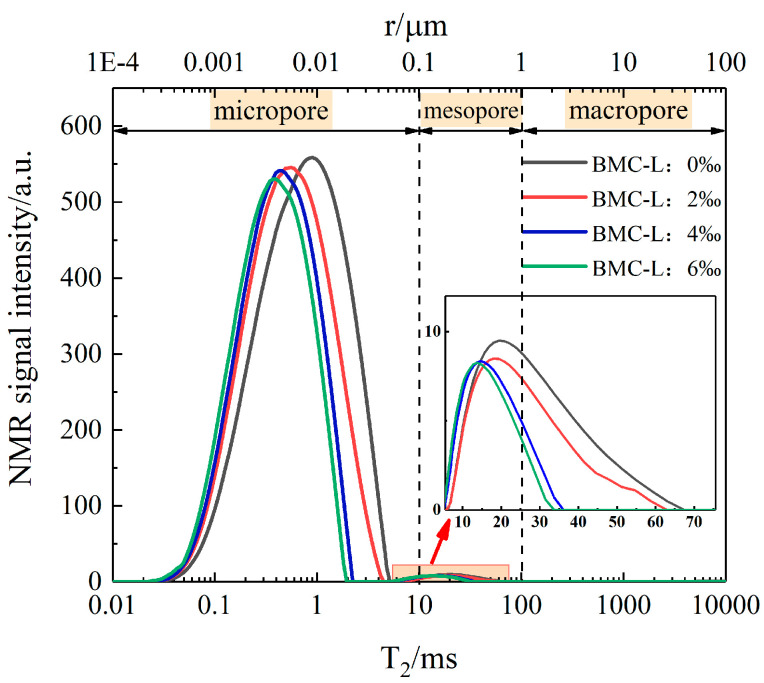
NMR *T*_2_ spectra of the slurry stone body with different BMC-L dosages.

Figure 11 displays the NMR *T*_2_ spectra of slurry stone bodies with varying BMC-L contents, where the upper axis represents the converted pore radius. The figure shows that as BMC-L dosage increases, the NMR signal from the slurry stone body specimens decreases, suggesting a reduction in internal pore volume and overall porosity. There are two peaks in the *T*_2_ spectra of the sample. The first peak *T*_2_ from left to right is mainly between 0.05 and 7.5 ms, and the corresponding pore size is 0.005–0.75 μm, indicating that the first peak is in the range of micropore pores. The second peak *T*_2_ is mainly in 7.5–68 ms, and the corresponding pore size is 0.075–0.68 μm, indicating that the second peak mainly contains micropores and mesopores, and the mesopores account for most of the pores. With the increase of BMC-L dosage, the peak area of the *T*_2_ spectra curve of the sample decreases continuously, and both peaks shift to the left, indicating that the internal pores of the sample decrease and the pore radius decreases. This occurs because polycarboxylate superplasticizers render the cement slurry more uniform, allowing the cement hydration products to distribute more evenly. This uniform distribution effectively fills the micropores and microcracks within the slurry, thereby reducing both the porosity and pore radius of the slurry stone body.

#### 3.7.2. Phase Composition

The X-ray diffraction (XRD) technique is a method to accurately identify the crystal structure and phase composition of materials by measuring the diffraction angle and intensity distribution of samples to X-rays. Widely utilized across various scientific and engineering disciplines, including physics, chemistry, and materials science, XRD has become an indispensable method for material structure analysis. The primary advantage of XRD is its capability to elucidate the crystal structure of a material and to identify and quantitatively analyze its phase composition. In cement-based material research, XRD is employed not only to determine the mineral composition of cement clinker but also to analyze the phase and structure of its hydration products. Consequently, XRD is pivotal in assessing the performance and optimizing formulations of cement-based materials.

**Figure 12:**
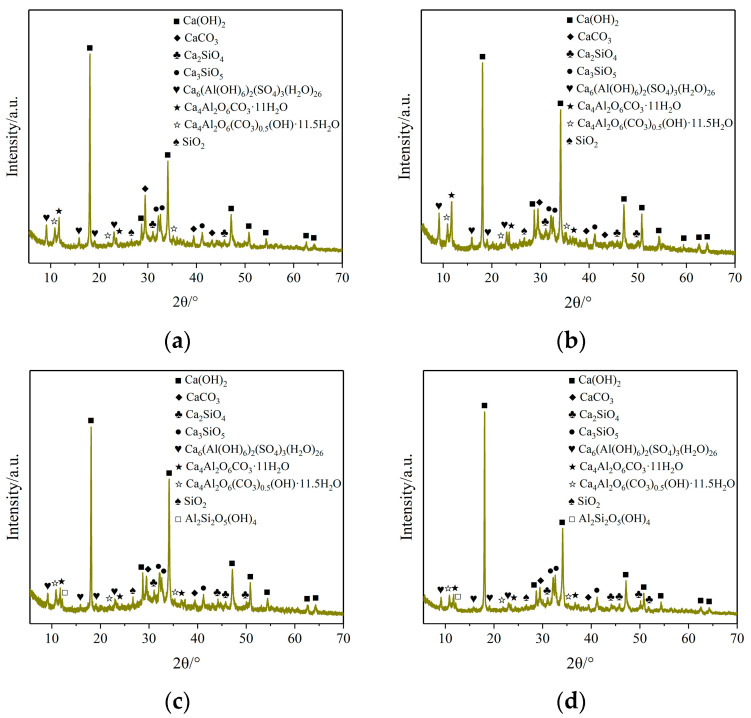
XRD patterns of slurry concretion with different BMC-L dosages: (**a**) BMC-L dosage: 0; (**b**) BMC-L dosage: 2‰; (**c**) BMC-L dosage: 4‰; (**d**) BMC-L dosage: 6‰.

To analyze the phase types and content changes in slurry stone bodies with varying BMC-L dosages, XRD was employed to test these variations. The XRD patterns of each slurry stone body are shown in Figure 12. Different hydration products correspond to different diffraction angles, and the heights of the diffraction peaks correspond to different levels of hydration product content. According to Figure 12, the hydration products remain consistent at BMC-L dosages of 0 and 2‰. At higher dosages of 4‰ and 6‰, a small quantity of kaolinite (Al_2_Si_2_O_5_(OH)_4_) begins to appear in the hydration products, as seen in Figure 12c,d. This may be due to the fact that a higher dosage of superplasticizer enhances its dispersion effect, thereby facilitating more complete hydration.

**Figure 13:**
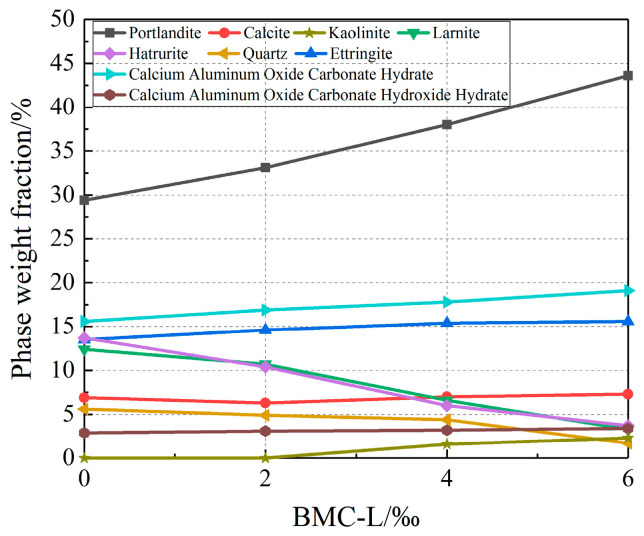
The effect of BMC-L dosage on the phase weight fraction of the slurry stone body.

To further investigate the variation in phase content of slurry stone bodies with BMC-L dosage, a quantitative analysis of their phase composition was conducted. The results of this analysis are presented in Table 6 and Figure 13. Figure 13 illustrates that portlandite is the predominant phase in the slurry stone composition, followed by ettringite and calcium aluminum oxide carbonate hydrate. As BMC-L dosage increases, the proportions of portlandite, ettringite, calcium aluminum oxide carbonate hydrate, and calcium aluminum oxide carbonate hydroxide hydrate in the slurry stone body’s phase composition rise, while those of larnite, hatrurite, and quartz diminish. This trend suggests that BMC-L enhances hydration reactions; higher dosages result in more complete hydration and greater formation of hydration products.

## 4. Conclusions

In this paper, varying dosages of polycarboxylate superplasticizer were incorporated into cement-based grouting materials. A comprehensive suite of tests and characterization techniques was employed to elucidate the impact of polycarboxylate superplasticizer on the density, viscosity, stability, and stone body mechanical properties and microstructure of the cement-based grouting materials. The following conclusions were drawn:

(1) Within the dosage range of 0 to 6 ‰, two types of polycarboxylate superplasticizers effectively lower slurry viscosity and enhance flowability. As the dosage of polycarboxylate superplasticizer increases, both the viscosity and the stone formation rate of the slurry decrease, though the stone formation rate remains within an acceptable limit. This effect occurs because the adsorption layer formed by the superplasticizer molecules on the cement particle surfaces minimizes direct particle contact, thereby reducing the internal friction and cohesion within the mixture;
(2) Polycarboxylate superplasticizers can significantly enhance the mechanical properties of the slurry stone body. As the dosage of BMC-L increases, there are noticeable improvements in the compressive strength, shear strength, and elastic modulus of the slurry. This enhancement is attributed to the superplasticizer’s ability to improve the dispersion of cement particles, resulting in a more uniform and dense cement slurry. Consequently, the hydration products formed after cement hydration are distributed more evenly, effectively filling the micropores in the slurry and reducing the formation of microcracks;
(3) Polycarboxylate superplasticizers can enhance the hydration of cement, thereby improving the microstructure of the slurry stone body and reducing both porosity and pore radius. This effect occurs because polycarboxylate superplasticizers facilitate the uniform dispersion of cement slurry and expand the hydration reaction area on the surface of cement particles. This improvement increases the yield of cement hydration products and promotes the formation of more uniform and denser structures.

## Figures and Tables

**Table 1:** Main chemical composition of test cement ^1,2^.

Chemical Composition	SiO_2_	Al_2_O_3_	Fe_2_O_3_	CaO	MgO	SO_2_
Value/%	21.32	4.31	3.38	61.26	2.47	2.55

^1^ specific surface area: 320 m^2^/kg. ^2^ Loss on ignition: 1.9%.

**Table 2:** Properties of superplasticizers used in the test.

	BMC-L	BMC-S
Appearance	Transparent liquid	White solid powder
solid content	38 ± 1.9%	-
pH	5.0 ± 1.0	7.0 ± 1.0
Chloride ion content	≤0.6 by weight	≤0.6 by weight
Total alkali content	≤10% by weight	≤10 by weight
Density	1.06 ± 0.02 g/cm^3^	-
Recommended dosage	3–10‰ by cement weight	1–15‰ by cement weight

**Table 3:** Specimen shear strength parameters.

Water–Cement Ratio	BMC-L Dosage/‰	c/MPa	φ/°
0.6:1	0	2.10	48.6
	2	2.32	50.3
	4	2.81	55.2
	6	3.16	63.1

**Table 4:** *T*_2_ surface relaxation strength values for reference [26].

$\rho_{2}$ (μm/ms)	Water	Clay	Solids
Water	0.000	0.010	0.003
Clay	0.010	0.010	0.003
Solids	0.003	0.003	0.000

**Table 5:** Comparison of common pore aperture division methods [27,28].

Reference	Micropore/μm	Mesopor/μm	Macropore/μm
De Quervain (1967)	<5	5–200	200–2000
Dubinin (1979)	(0.0012–0.0014)–(0.003–0.0032)	(0.003–0.0032)–(0.2–0.4)	>(0.2–0.4)
IUPAC (Gregg and Sing 1982)	<0.002	0.002–0.05	>0.05
Klopfer (1985)	<0.1	0.1–1000	>1000
DIN66131 (1993)	<0.002	0.002—0.05	>0.05
Kodikara et al. (1999)	1–30	—	10–1000
He Yudan et al. (2005)	<10	>10	
Lonoy (2006)	10–50	50–100	>100
Yan Jianping et al. (2016)	<0.1	0.1–1	>1
Fang Tao et al. (2017)	<0.1	0.1–1	1–5

**Table 6:** Physical phase weight fractions of different BMC-L doped slurry stone bodies, %.

Phase Name	BMC-L Doasge/‰			
	0	2	4	6
Portlandite(Ca(OH)_2_)	29.4	33.1	38	43.6
Calcite(CaCO_3_)	6.9	6.3	7	7.3
Ettringite(Ca_6_(Al(OH)_6_)_2_(SO_4_)_3_(H_2_O)_26_)	13.5	14.6	15.4	15.6
Larnite(Ca_2_(SiO_4_))	12.4	10.7	6.6	3.3
Hatrurite(Ca_3_(SiO_4_)O)	13.7	10.4	6	3.7
Quartz(SiO_2_)	5.6	4.9	4.4	1.7
Calcium Aluminum Oxide Carbonate Hydrate(Ca_4_Al_2_O_6_CO_3_·11H_2_O)	15.6	16.9	17.8	19.1
Calcium Aluminum Oxide Carbonate Hydroxide Hydrate(Ca_4_Al_2_O_6_(CO_3_)0.5(OH)·11.5H_2_O)	2.9	3.1	3.2	3.4
Kaolinite(Al_2_Si_2_O_5_(OH)_4_)	0	0	1.6	2.3

## Data Availability

The original contributions presented in the study are included in the article, further inquiries can be directed to the corresponding author.
